# Neutrophil elastase cleavage of the gC1q domain impairs the EMILIN1-α4β1 integrin interaction, cell adhesion and anti-proliferative activity

**DOI:** 10.1038/srep39974

**Published:** 2017-01-11

**Authors:** Orlando Maiorani, Eliana Pivetta, Alessandra Capuano, Teresa Maria Elisa Modica, Bruna Wassermann, Francesco Bucciotti, Alfonso Colombatti, Roberto Doliana, Paola Spessotto

**Affiliations:** 1 Experimental Oncology 2, Department of Translational Research, CRO-IRCCS, National Cancer Institute, Aviano 33081, Italy

## Abstract

The extracellular matrix glycoprotein EMILIN1 exerts a wide range of functions mainly associated with its gC1q domain. Besides providing functional significance for adhesion and migration, the direct interaction between α4β1 integrin and EMILIN1-gC1q regulates cell proliferation, transducing net anti-proliferative effects. We have previously demonstrated that EMILIN1 degradation by neutrophil elastase (NE) is a specific mechanism leading to the loss of functions disabling its regulatory properties. In this study we further analysed the proteolytic activity of NE, MMP-3, MMP-9, and MT1-MMP on EMILIN1 and found that MMP-3 and MT1-MMP partially cleaved EMILIN1 but without affecting the functional properties associated with the gC1q domain, whereas NE was able to fully impair the interaction of gC1q with the α4β1 integrin by cleaving this domain outside of the E933 integrin binding site. By a site direct mutagenesis approach we mapped the bond between S913 and R914 residues and selected the NE-resistant R914W mutant still able to interact with the α4β1 integrin after NE treatment. Functional studies showed that NE impaired the EMILIN1-α4β1 integrin interaction by cleaving the gC1q domain in a region crucial for its proper structural conformation, paving the way to better understand NE effects on EMILIN1-cell interaction in pathological context.

Studies in recent decades have established the constituents of the extracellular matrix (ECM) as dynamic structures that can profoundly influence diverse aspects of cellular behaviour and functions^1^,^2^. Integrins, a large family of αβ heterodimeric cell membrane receptors, have emerged as key sensory molecules that translate chemical and physical cues from the ECM into biochemical signals that regulate cell adhesion and shape, migration and tumour cell metastases, differentiation, proliferation, and survival^3^,^4^. EMILIN1 is an ECM glycoprotein containing the amino-terminal EMI domain, a cysteine-rich sequence of approximately 80 amino acids, and a gC1q domain at the carboxyl-terminal end^5^,^6^,^7^. EMILIN1 belongs to the C1q/TNF superfamily whose hallmark is a homo- and hetero-trimeric gC1q domain. The members of this superfamily play a broad spectrum of functions in innate immunity, insulin sensitivity and collagen hexagonal lattice organization to name a few^8^,^9^. EMILIN1 is strongly expressed in the blood and lymphatic vessels and in the connective tissues of a wide variety of organs^10^,^11^,^12^,^13^. It is involved via the EMI domain in the maintenance of blood vascular cell morphology and function^14^; in addition, EMILIN1 promotes adhesion and migration^15^,^16^ and controls cell proliferation^13^,^17^ through its gC1q domain. So far, among the gC1q domains of several other ECM components, EMILIN1 gC1q is the only one capable of interacting with the α4β1 integrin^15^,^16^,^17^,^18^, which is predominantly expressed on the surface of hemopoietic cells, working as a receptor to allow adhesion to blood vessel wall^19^ or extracellular matrix constituents^20^. The interaction between α4β1 and EMILIN1 gC1q is particularly efficient because even very low ligand concentrations provide very strong adhesion and migration^15^,^16^. The residue E933 located by NMR in a unstructured loop at the apex of the gC1q homotrimer, plays a crucial role in specifically engaging α4β1 integrin^18^. Mutagenesis of several residues around E933 indicated that the α4β1 integrin binding site involved exclusively this residue^18^. However, recent results showed that the EMILIN1-α4β1 interaction results likely different respect to conventional α4β1 binding to short linear peptide consensus sequences that represents a common feature of ECM integrin ligands^21^,^22^. In fact, regions outside the main ligand loop are implicated in the interaction mechanism (Capuano *et al*. in preparation). Targeted inactivation of the *emilin1* gene in the mouse, in addition to a lymphatic phenotype^12^,^23^, induces an increased thickness of epidermis and dermis^13^. The interaction between EMILIN1 with α4β1 (expressed on fibroblasts) and the closely related α9β1 (expressed on keratinocytes) provides an important external regulatory signal for the maintenance of a correct homeostasis between proliferation and differentiation in the skin. All these findings highlight the uniqueness of EMILIN1 for its integrin receptors: differently from what happens when other ECM ligands bind to α4 or α9^24^,^25^,^26^, the signal transduced by EMILIN1 through integrins has net anti-proliferative effects.

We previously demonstrated that among the proteolytic enzymes released in the tumour microenvironment neutrophil elastase (NE) was the most effective cleaving enzyme able to fully impair the regulatory function of EMILIN1^27^. EMILIN1 was fragmented in sarcomas and ovarian cancers and likely was associated to a higher proliferation rate in these tumors^27^. Moreover, EMILIN1 digestion by NE with the consequent weakness of the intercellular junctions of lymphatic endothelial cells was correlated to the acute phase of acquired lymphoedema^28^. EMILIN1 fragmentation results in a condition similar to that of the ablated molecule in *Emilin1*^−/−^ mice leading to uncontrolled cell proliferation and lymphatic phenotype^12^,^13^,^23^. It is reasonable to argue that EMILIN1 digestion by the action of proteolytic enzymes can be a pathogenic mechanism leading to the loss of functions associated to the functional gC1q domain. Using a proteomic approach on components of vasculature, other authors have proposed EMILIN1 as a potential candidate substrate also for MMP-3, -9 and MT1-MMP^29^. Considering that gC1q is involved in most of the crucial properties of EMILIN1, we aimed to investigate if proteolytic enzymes, in particular NE and the proposed MMPs, were able to efficaciously cleave this functional domain.

## Results

### EMILIN1 is partially cleaved by MMPs

**Figure 1:**
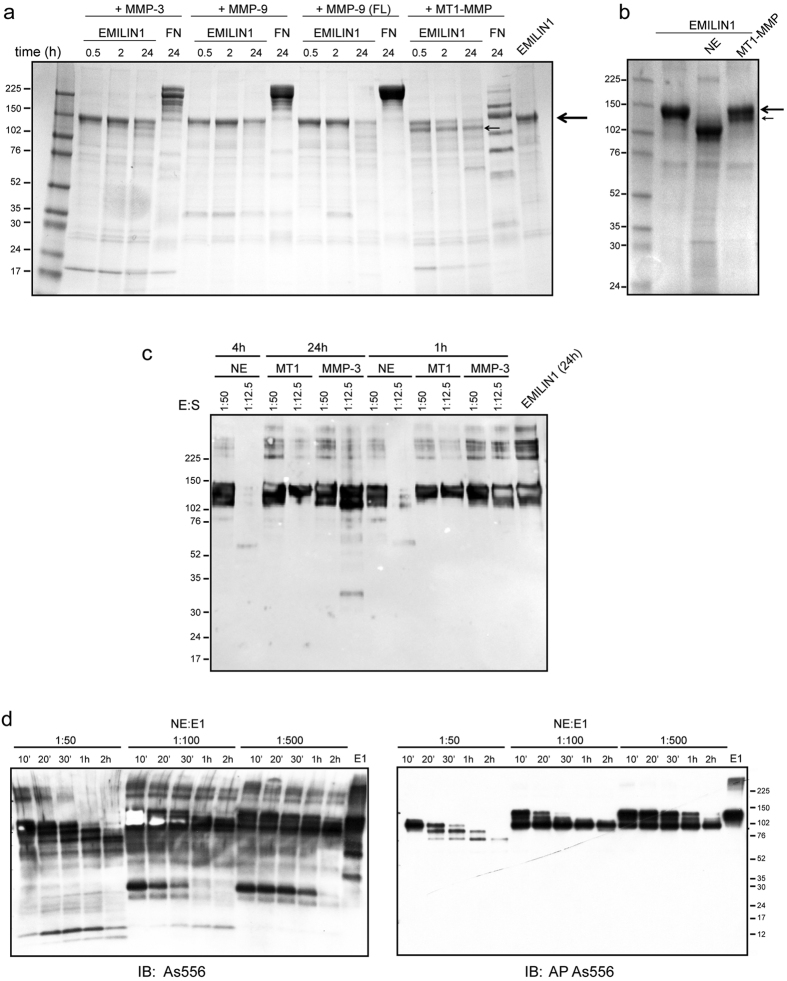
Enzymatic action of MMPs and NE on EMILIN1. (**a**) EMILIN1 was incubated with the recombinant catalytic domain of MMP-3, MMP-9 or MT1-MMP (MT1) at a 1:4 (E:S) molar ratio and with the full length (FL) form of the recombinant MMP-9 at a 1:20 molar ratio at 37 °C for 30 min, 2 h or 24 h. The cleavage products were detected by Coomassie staining. FN was used as a positive control of cleavage. (**b**) Coomassie staining of EMILIN1 samples incubated with NE (1:50, 1 h) or MT1-MMP (1:4, 24 h). Black arrows and smaller black arrows indicate the intact band and fragmented band of EMILIN1, respectively. (**c**) Western blotting analysis of EMILIN1 samples incubated with MMP-3 and MT1-MMP (MT1) or NE at times and doses as indicated. Membranes were probed with polyclonal antibodies against EMILIN1 (As556). (**d**) Western blotting analysis of dose and time-dependent EMILIN1 fragmentation by NE. Reactivity for As556 (left) and AP As556 (right) is displayed.

To test whether MMPs were able to cleave EMILIN1, recombinant EMILIN1 was incubated with the recombinant catalytic domains of MT1-MMP, MMP-3 or MMP-9 at very high molar ratios (1:4–E [Enzyme]:S [Substrate]) and also with the full length form of the recombinant MMP-9 at a ratio of 1:20 - E:S at 37 °C. The digestions were carried out for different times as indicated. Under these conditions, Coomassie staining revealed a slight cleavage activity especially by MT1-MMP after a 24 h incubation with the reduction of the principal band (Fig. 1a). EMILIN1 digested with MT1-MMP formed a characteristic band migrating very close to the principal intact EMILIN1 band and already visible after 0.5 h of incubation (Fig. 1a, smaller arrow). A similar band was detectable also in the presence of MMP-3 but only after much longer incubation times. MMP-9 was ineffective. Fibronectin (FN), used as positive control of digestion and incubated with the enzymes at the same molar ratio used for incubation with EMILIN1, was cleaved by all MMPs with different pattern and extent (Fig. 1a). To further confirm whether MT1-MMP and MMP-3 cleaved EMILIN1, the full-length enzymes were transiently transfected into HEK293 cells (Supplemental Fig. S1a). After 24 h from transfection recombinant EMILIN1 was added to the cell cultures and the resulting conditioned media collected following different incubation times. Both MMP-3 and MT1-MMP show a very limited activity on EMILIN1 generating a similar pattern of fragmentation: two large preponderant bands were detectable and migrated very close to the main band (about 140 kDa) corresponding to the intact EMILIN1 still present after long incubation times (Supplemental Fig. S1b, red arrow heads) and two minor smaller fragments were found at about 60 kDa (Supplemental Fig. S1b, red arrows). An additional band of 50 kDa was produced only by the MMP-3 proteolytic action (Supplemental Fig. S1b). The intensity of the slower migrating bands (250 kDa), corresponding to EMILIN1 dimers^30^, progressively decreased in both MMP conditioned media, indicating that the proteolytic action targeted also EMILIN aggregates (Supplemental Fig. S1b). To verify MMP-9 activity on EMILIN1 we used supernatants from human osteoclasts since these cells very efficiently and constitutively produce this enzyme which is present also in the activated form (Supplemental Fig. S1c). No fragmentation bands were detectable following a 24 h incubation with recombinant EMILIN1 (Supplemental Fig. S1d), confirming the results obtained using the recombinant enzyme (Fig. 1a). Comparing MMP and NE proteolytic action on EMILIN1, we observed that NE displayed a much higher affinity for this substrate since lower amounts of NE (1:50 - E:S) were able to cleave EMILIN1 even earlier: after 1 h, the intact band of EMILIN1 was no more detectable, whereas after a 24 h incubation with MT1-MMP the band of intact EMILIN1 was still present even if slightly reduced (Fig. 1b). The fragmentation pattern was analyzed also in WB with a rabbit antiserum against EMILIN1 (As556) to detect the specificity of fragmented bands and to compare MMPs and NE activity. Again lower doses and earlier times of incubation were sufficient to obtain EMILIN1 fragmentation by NE (Fig. 1c). Moreover, after 24 h MMPs were unable to completely cleave EMILIN1 since the band corresponding to the intact form was clearly still detectable (Fig. 1c). NE activity was very efficient in EMILIN1 cleavage: we performed dose and time dependent experiments, detecting fragmentation patterns by both As556 antibody and the same antiserum subjected to affinity purification to recognize only the gC1q domain (AP As556). The results indicated that in the presence of NE the loss of EMILIN1 intact band as well as that of fragments containing immunodetectable gC1q was rapid even at low enzyme concentrations (Fig. 1d). NE derived products still detectable with As556 lost very early positive staining for AP As556, suggesting that NE action likely affects more efficaciously the immunogenicity of the gC1q domain (Fig. 1d). From these experiments we can conclude that in this system the activity of the tested MMPs on EMILIN1 substrate is very low, if not absent at least for MMP-9, as only at high enzyme concentrations and long incubation times a minor fraction of the molecule was fragmented, whereas NE completely digested EMILIN1 at low concentrations and short incubation time.

### MMP proteolytic action does not interfere with EMILIN1 adhesive and anti proliferative properties

**Figure 2:**
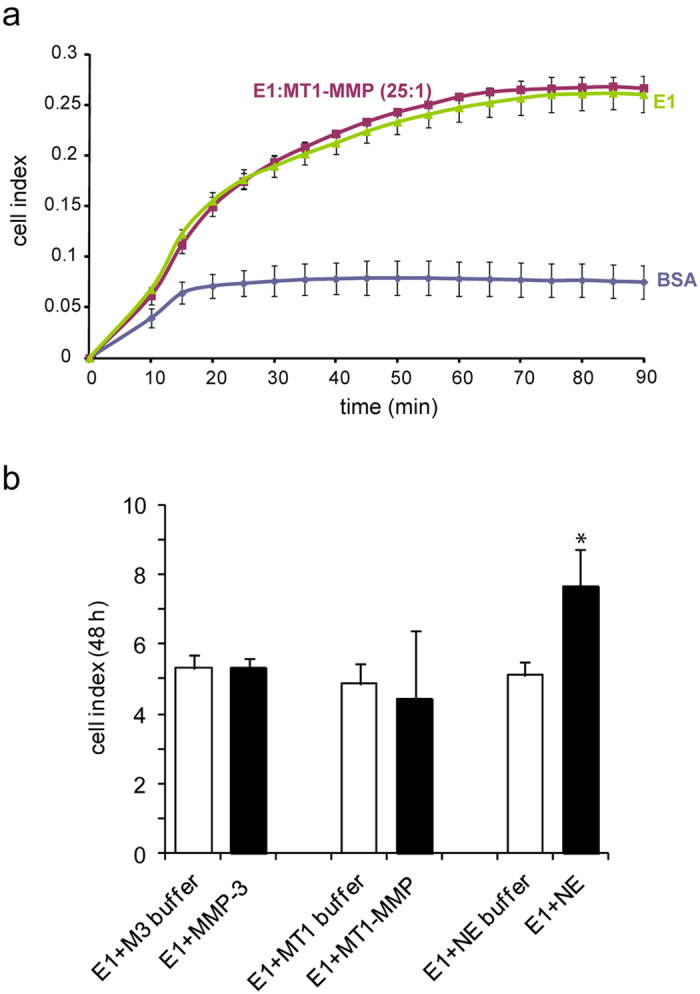
MT1-MMP treatment does not impair EMILIN1 functional properties. (**a**) Dynamic monitoring of SKLMS1 cell attachment in response to the effect of MT1-MMP on EMILIN1 (E1) cleavage measured with the XCELLigence instrument and expressed as the cell index. A 1:25 (E:S) molar ratio was used for a 24 h incubation. The wells were finally coated with 10 μg/ml of EMILIN1 (incubated or not with MT1-MMP). The data shown is the mean ± SD from n = 3 experiments with n = 6 replicates. (**b**) SKLMS1 cell proliferation 48 h after plating. The cell index of dynamic monitoring calculated as the mean ± SD from n = 3 experiments with n = 6 replicates is reported. The wells were coated with EMILIN1 (10 μg/ml) treated with MMP-3, MT1-MMP (1:25–E:S) or NE (1:200–E:S) or incubated in the corresponding (M3, MT1, NE) buffer for 24 h. *P < 0.05.

We have previously demonstrated that the anti-proliferative role of EMILIN1 is impaired only by NE cleavage^27^, suggesting that very likely the integrity of the regulatory domain gC1q is lost in the presence of this enzyme. To verify if the cleavage of EMILIN1 by MMPs could modify the functional properties of the protein, we first examined if cells were still able to attach to the fragmented protein. EMILIN1 was first incubated with MT1-MMP for 24 h, then the products of digestion were distributed into 96-plate wells and SKLMS1 cells, that express α4β1 integrin^27^, were added to the wells and allowed to adhere. We did not notice any reduction in cell adhesion after digestion with MT1-MMP (Fig. 2a). Therefore, even if MT1-MMP partially cleaved EMILIN1, this proteolityc action did not affect the site of interaction with α4β1 integrin. Alternatively, as MT1-MMP was unable to fully digest the total amount of EMILIN1 in its intact form, as detected by gel and WB analyses (Fig. 1 and Supplemental Fig. S1), the uncleaved protein was sufficient to fully sustain the adhesion of SKLMS1 cells to EMILIN1. Accordingly, no variation on proliferation regulated by EMILIN1 was detected after its incubation with MT1-MMP (Fig. 2b). On the contrary, SKLMS1 proliferated on NE-treated EMILIN1 more than cells grown on intact EMILIN1 (Fig. 2b), as we previously showed^27^.

### NE is able to digest gC1q

**Figure 3:**
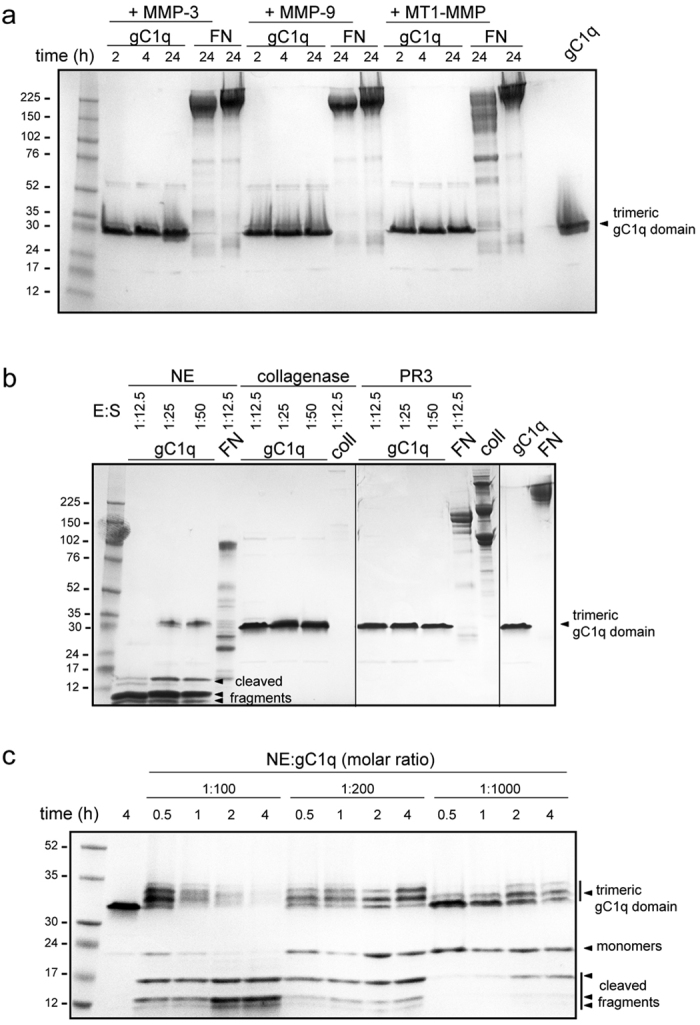
NE cleaves EMILIN1 functional domain. Coomassie staining of gC1q digestion curves by MMPs, collagenase, PR3, and NE. The resulting mixtures were loaded on SDS PAGE gels without boiling to prevent the dissociation of the trimeric gC1q into monomers. Representative full length gels (**a** and **b**) or the lower part (**c**) are shown. (**a**) gC1q is not cleaved by MMPs even at high enzyme concentration. Incubations at different times (2, 4 and 24 h) were performed with a molar ratio of 1:37 (E:S). FN was used as positive control of cleavage. (**b**) Collagenase and PR3 were added to gC1q for 1 h at different doses as indicated. Collagen type I (coll) and FN were used as positive control of cleavage. No digestion fragments are detected. Vertical lines indicated cropped gels derived from independent runs. (**c**) Dose- and time-dependent digestion curves of gC1q by NE show that increasing concentration of NE and prolonged time of incubation produce the appearance of cleaved fragments and the formation of a slower migrating form of gC1q trimer, as indicated by arrow heads. Note that above all at very low doses of the enzyme the stability of the gC1q homotrimer is highly compromised.

Considering that a specific cleavage within the functional domain could be a reasonable mechanism to impair EMILIN1 binding capabilities, to verify a direct proteolytic action we focused our attention on the gC1q domain. The recombinant gC1q domain was incubated with different enzymes and the resulting mixtures loaded on SDS PAGE gels. MMPs did not display any effect on gC1q even at long time of incubations (Fig. 3a). We checked if other serine proteinases such as proteinase-3 (PR3) which shares high homology with NE and is able to digest the full-length EMILIN1^27^, could display a proteolytic activity on gC1q. We did not detect any fragmentation suggesting that PR3 cleaves EMILIN1 outside the gC1q domain (Fig. 3b). Similarly, also collagenase did not digest gC1q domain (Fig. 3b). On the contrary, the incubation with NE produced a time and dose-dependent gC1q fragmentation (Fig. 3b,c). As shown in Fig. 3c, the gC1q monomers became visible over time together with a concomitant increase of two bands migrating between 12 and 17 kDa. Depending on dose and time, almost all the gC1q trimeric structures were converted in digested fragments by NE (Fig. 3b,c, arrow heads). Another characteristic effect of the NE enzymatic action was represented by the migratory changes of the trimeric form of gC1q: the domain has a compact structure that confers a characteristic faster migration in SDS gel, resulting in an apparent molecular weight of about 33 kDa, much different from the calculated 52 kDa. NE was apparently able to produce a progressive “unwinding” of the complex with a decompacting effect of the trimer and the formation of mixed slowly migrating forms (Fig. 3c). The different migration could reflect a subtle modification of the trimeric domain folding due to the cleavage of one, two or three monomers still remaining associated in the trimer via the known strong hydrophobic interactions^18^. At low NE concentrations the unwound trimers partially released intact monomers migrating at about 20 kDa, whereas at higher concentration and/or prolonged incubation the trimers completely dissociated and all the monomers were cleaved (Fig. 3c). Moreover, using a very low E:S molar ratio (1:1000) (comparing to that of MMPs that was 1:37) we were able to detect the unwinding trimeric form and the digestion fragments already at 1 h of incubation (Fig. 3c), indicating that NE had a very high affinity for gC1q. This data allowed us to conclude that NE, among the enzymes analysed, was the only one able to display a relevant proteolytic activity on gC1q.

### Identification of the cleavage site on gC1q

**Figure 4:**
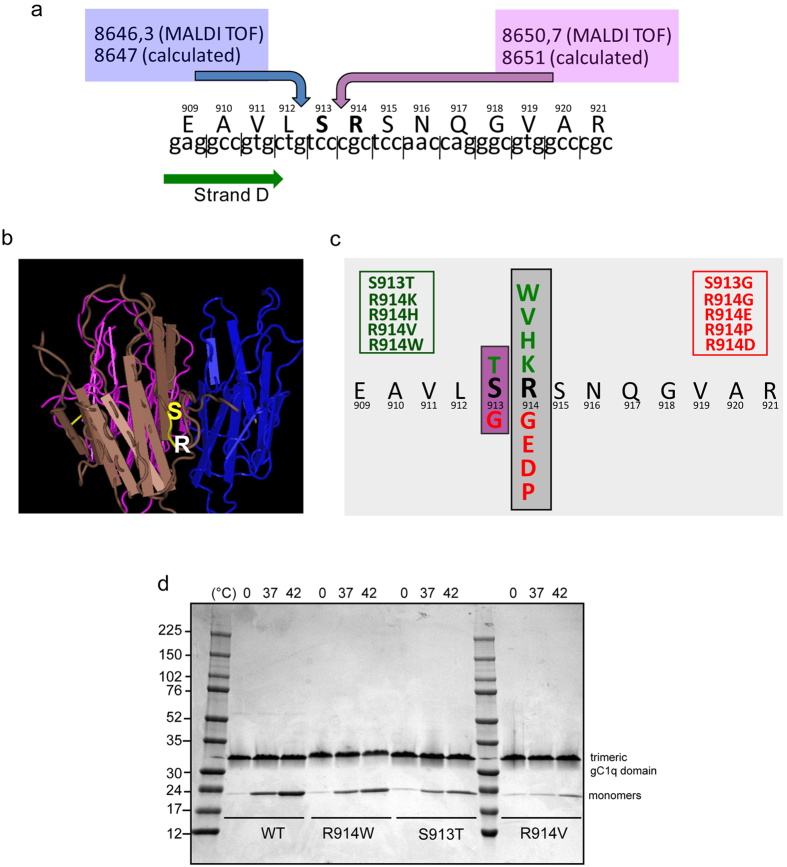
Identification of the possible NE cleavage site on gC1q. (**a**) Schematic representation of gC1q primary structure with the two possible cleavage sites emerged from MALDI TOF analysis, one placed between S913 and R914 (8651calculated MW, blue arrow) and a second between L912 and S913 (8647 calculated MW, red arrow). (**b**) NMR model of the gC1q domain where the cleavage site is highlighted in yellow to indicate its good exposure to the solvent to favor the interaction with NE. (**c**) gC1q mutants produced with a site-directed mutagenesis approach. Soluble mutants are indicated in green whereas the insoluble in red. (**d**) Coomassie staining of the melting transition profile of the gC1q domain. Ten μg of WT and mutants of gC1q domain were incubated in sample buffer for 1 h at the indicated temperature and then separated in a 4–20% gradient SDS-PAGE. Mutants show a melting pattern comparable to that of the WT domain.

**Figure 5:**
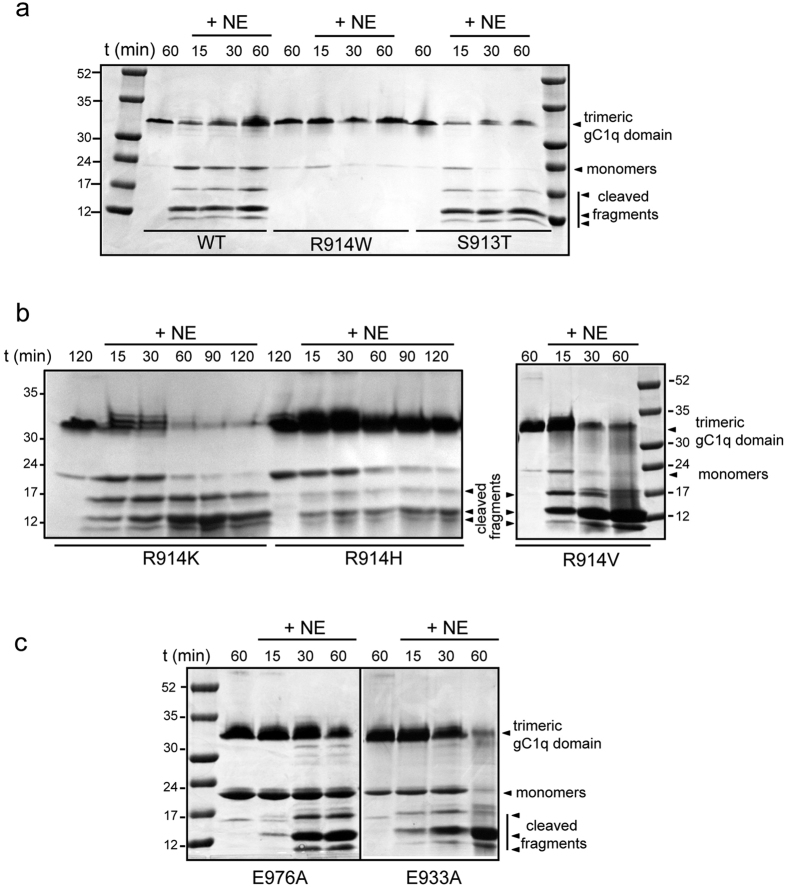
R914W gC1q mutant is resistant to NE enzymatic degradation. Degradation pattern (Coomassie staining of representative gels, lower part) of gC1q mutants (**a)** R914W and S913T; (**b**) R914K, R914H and R914V; (**c**) E976A and E933A) after incubation with NE at 1:200 (E:S) molar ratios at different times as indicated. Note that all the mutants except R914W are well fragmented as well as WT gC1q after very short times of incubation.

According on the MEROPS peptidases database (http://merops.sanger.ac.uk) and a recent analysis based on the proteomic PICP approach^31^, NE has a quite loose active site specificity, lacking a canonical cleavage site with each subsite accommodating several residues (Supplemental Fig. S2). Thus, we followed a mutagenesis analysis approach to experimentally determine the precise cleavage sites. First, to restrict as much as possible the cleavage site the main product derived from gC1q digested with NE and migrating at the apparent molecular weights ranging from 12 to 17 kDa were gel purified and analyzed by MALDI TOF analyses. The resulting two main peaks (data not shown) with a MW of 8650,7 and 8646,3 Da matched almost perfectly to virtual fragments with a calculated mass of 8651 Da for cleavage between S913 (P1) and R914 (P1′), or 8647 for cleavage between L912 (P1) and S913 (P1′) (Fig. 4a). These hypothetical cleavage sites are properly exposed to solvent as shown by NMR model (Fig. 4b). Thus, several mutants of R914 and S913 were produced as recombinant gC1q domain with the aim to impair NE recognition and cleavage (Fig. 4c). The correct global folding of the soluble mutants (four for R914 position and one for S913 position) was assessed by solubility and thermostability assays as described^18^ (Fig. 4d) and then subjected to NE digestion. In line with the weak consensus cleavage site of NE, mutants S913T, R914H and R914V as well as the wild type form were digested (Fig. 5a,b); the mutant R914K was digested even more efficiently, indicating a positive gain when lysine is introduced in P1′ (Fig. 5b). On the contrary, the mutant R914W displayed a significant resistance to NE digestion: more than 90% of the trimeric polypeptide appeared intact after 1 h treatment (Fig. 5a). To be noted, according to the NE specificity matrix reported in MEROPS Database, Tryptophan frequency in position P1′ is close to zero (2/483) (Supplemental Fig. S2). Several other mutants distant from the hypothesized cleavage site, including the mutation E993A that impairs the interaction of gC1q with α4β1 integrin^18^, displayed a NE sensitivity comparable to that of the wild type (Fig. 5c).

### The R914W gC1q mutant retains its functions after NE treatment

**Figure 6:**
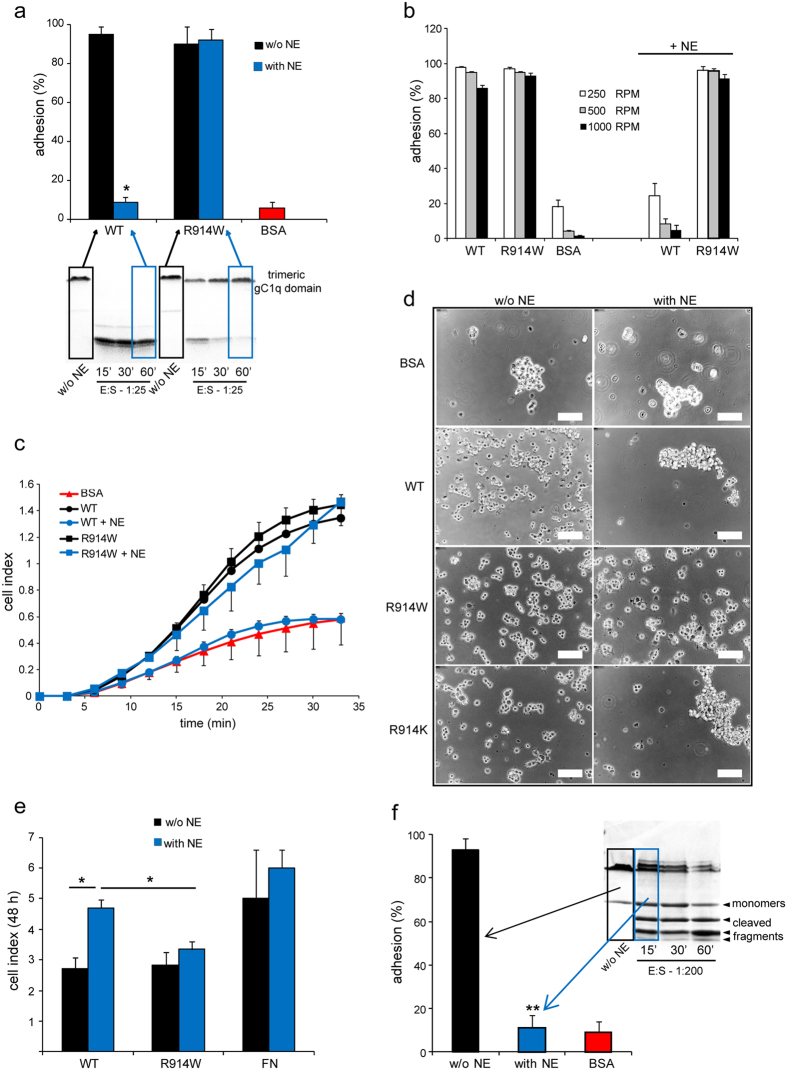
R914W gC1q mutant retains functional properties after NE treatment. (**a**) Adhesion (CAFCA assay) of Jurkat cells on WT or R914W gC1q preincubated (with NE) or not (w/o NE) with NE. The E:S molar ratios and incubation times are “boxed” in the corresponding gel at the bottom of the graphic indicating the status (fragmented or not) of the resulting gC1q used to coat the wells (10 μg/ml). (**b**) Differential centrifugal forces applied in the reverse direction during the second centrifugal step of CAFCA assay (see Materials and Methods) to allow the removal of the unbound/weakly bound cells under controlled conditions. The relative Jurkat cell adhesion strengths to WT and R914W gC1q preincubated or not with NE at the same conditions applied in (**a**) are reported. R914W gC1q incubated with NE allowed Jurkat cell adhesion at each reverse centrifugal force applied. (**c**) Dynamic monitoring of SKLMS1 cell attachment in response to the effect of NE on WT and R914W gC1q cleavage measured with the XCELLigence instrument and expressed as cell index. Dose and incubation time are the same as used in (**a**). The wells were coated with 10 μg/ml of gC1q (incubated or not with NE). (**d**) Morphological appearance of SKLMS1 adhering (30 min) on WT or gC1q mutants treated or not with NE (1:25–E:S, 1 h). BSA was used as control. (**e**) Proliferation of SKLMS1 cells plated on gC1q (WT or R914W mutant) (10 μg/ml) treated with NE (1:25–E:S, 1 h). The cell index after 48 h of dynamic monitoring calculated as the mean ± SD from n = 3 experiments with n = 6 replicates is reported. *P < 0.05. (**f**) Adhesion of Jurkat cells on WT gC1q preincubated or not with NE. The E:S molar ratio and incubation time are “boxed” in the corresponding gel next to the graphic. Data are expressed as the means ± SD of n = 3 independent experiments with n = 6 replicates. **P < 0.001.

The EMILIN1 gC1q domain promotes the firm adhesion of Jurkat cells via α4β1 integrin interaction^15^. Here, we used this model as a functional read out to evaluate the effect of NE on the adhesion properties of the gC1q domain. As reported in Fig. 6(a and b), Jurkat cells adhered as expected to the WT gC1q, but this capability was completely lost after incubation of the ligand with NE. On the other hand, treatment of R914W with NE did not impair cell adhesion, indicating that the impairment by NE was due exclusively to gC1q fragmentation and not to a possible activity on different cell components. To better define the effect of gC1q adhesion properties differential centrifugal forces were applied in the second centrifugal step of the CAFCA assay (see Materials and Methods), where the reverse direction allowed for removal of the unbound/weakly bound cells under controlled conditions, in order to estimate the relative Jurkat cell adhesion strengths to WT and R914W gC1q treated or not with NE. The mutant R914W gC1q incubated with NE was still able to bind cells even at high force used in the reverse direction (1,000 RPM), whereas WT gC1q digested by NE bound cells very weakly even at low detaching forces (250 RPM) (Fig. 6b). Moreover, we monitored in real time if the R914W gC1q was capable to support cellular spreading after NE treatment. Of note, SKLMS1 cells spread well on R914W gC1q also after incubation with NE (Fig. 6c,d). It is known that the interaction between gC1q and α4β1 integrin has an inhibitory effect on cell proliferation^13^. To verify if NE digestion could affect this property we performed a proliferation assay using SKLMS1 cells. When the WT gC1q was treated with NE the anti-proliferative regulatory properties were strongly affected (Fig. 6e). On the contrary, the NE treated R914W mutant maintained a strong inhibitory effect on SKLMS1 proliferation. Moreover, a low NE:WT gC1q molar ratio and a very short time of incubation were sufficient to abolish the binding properties, even if the digestion was only partial after 15 min, with apparently a consistent amount of trimeric form still present (Fig. 6f). However, the migration pattern indicated that the majority of the trimers were unwound after NE treatment, suggesting that the conformational properties of the gC1q trimeric form were absolutely crucial for a proper integrin interaction. Taken together these results demonstrate that NE cleaved specifically the gC1q domain between S913 and R914, and that the fragmented gC1q lost its capacity to support both cell adhesion and cell proliferation.

**Figure 7:**
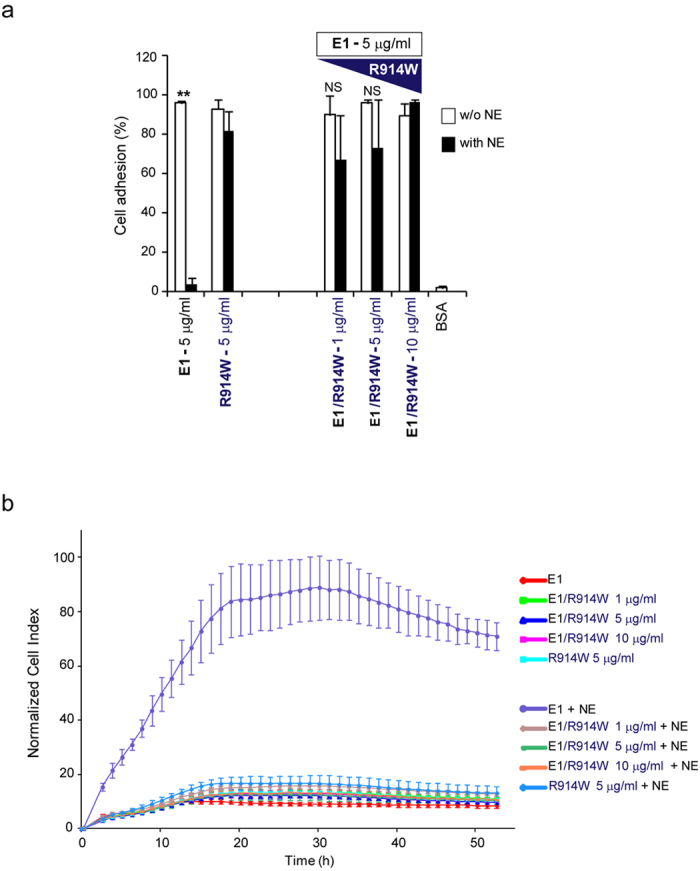
R914W gC1q mutant is able to fully compete in cell adhesion and proliferation assays when used as agonist. In adhesion (Jurkat cells, CAFCA assay, (**a**) and proliferation (SKLMS1 cells, dynamic monitoring by XCelligence instrument, (**b**) assays R914W gC1q mutant was present as an agonist at different doses as indicated. The mixtures (R914W gC1q mutant at different doses and EMILIN1, E1 at 5 μg/ml) were preincubated for 1 h with NE or not (w/o NE) and the resulting products used to coat the wells. Data are expressed as the means ± SD of n = 3 independent experiments with n = 6 replicates. **P < 0.001; NS, not significant.

To further support our hypothesis we performed cell adhesion as well as proliferation assays using the full-length EMILIN1 together with the R914W gC1q mutant at different doses as an agonist. We determined that the R914W gC1q mutant was able to fully compete in cell adhesion after NE treatment (Fig. 7a). Furthermore, in the proliferation assay the presence of the R914W mutant that is not digested by NE was able to suppress cell proliferation (Fig. 7b).

## Discussion

EMILIN1 is involved in cell adhesion, migration and skin proliferation homeostasis via interaction of its C-terminal gC1q domain with the α4β1 integrin. Proteases secreted by infiltrated neutrophils are able to fragment EMILIN1 in lymphoedematous tissues. This degradation is abolished by the NE specific inhibitor sivelestat but not by the MMPs broad-spectrum inhibitor GM6001^28^. In the present study we demonstrated that the functional properties of EMILIN1 were impaired only by enzymes able to digest gC1q and that NE is the only one among those we tested that specifically cleaves the EMILIN1 regulatory gC1q domain. MMPs activity on recombinant EMILIN1 *in vitro* was very limited if compared to that observed with NE. However, MMPs did not digest the recombinant gC1q domain that displayed resistance also to collagenase and PR3. Accordingly, MMPs were not able to impair adhesion nor proliferation of cells plated on EMILIN1 after enzymatic treatment. By a proteomic approach it has been showed that EMILIN1 from arterial tissue could be a substrate for MMP-3, MMP-9 and MT1-MMP^29^. Thus, the proteolytic action of MMPs could be fundamental in some pathological conditions affecting vessels, likely contributing to weakening of the vascular wall via their capacity to digest fundamental structural ECM components of elastic fibers. It will be interesting in the future to functionally investigate how crucial the EMILIN1 structural integrity is in the blood vascular context.

EMILIN1 cleavage by NE is critical in the regulation of cell proliferation. In the tumor microenvironment NE is likely the inflammatory enzyme displaying a proteolytic action on gC1q, thus impairing its integrity and then its suppressor role. NE is a neutrophil-derived serine proteinase with broad substrate specificity, including most components of the ECM such as elastin, collagens, laminin, entactin, and fibronectin^32^. In addition, a key role for NE in cleaving and either activating or inactivating cytokines and growth factors has been shown^33^,^34^. NE promotes cellular proliferation in several, different lung and breast cancer cell lines and in fibroblasts, as well^35^,^36^,^37^. In tumour cells NE accomplishes this by entering endosomes via the classic clathrin pit-mediated endocytosis, and then targeting intracellular substrates within the cytosol, such as IRS-1, a key mediator of PI3K signaling^36^,^38^. In our study we describe an indirect modality used by NE to favour cell proliferation.

The cleavage site within the gC1q domain lies between S913 and R914 residues and the mutant R914W was fully resistant to the NE proteolytic action and worked as agonist very well. This mutant was instrumental to demonstrate in a cellular context that the loss of gC1q-integrin interaction in the presence of NE was determined exclusively by the gC1q cleavage and not by other possible NE targets present in the milieu.

The EMILIN1 gC1q domain has been identified as a fundamental component of the whole molecule, both from a structural and a functional viewpoint^7^: it is the domain able to initiate and drive the homo-trimerization of the protein and it plays regulatory properties through the interaction with the α4/α9β1 integrins^13^,^15^,^16^,^23^. The α4β1 binding site is located in a flexible loop at the apex of the trimeric structure and it is absolutely dependent on the E933 residue^18^. In addition to the E933 residue it appears that also other regions of the gC1q domain are important for the integrin recognition (Capuano *et al*., in preparation), suggesting that the global folding of the gC1q is necessary for its full activity. The present data is in agreement with this concept: by partial digestion of gC1q with low concentrations and/or short times of incubation with NE an heterogeneous mix of unwound trimers was obtained, possibly due to the cleavage of one, two or three monomers still associated into trimers. These mixed products did not support any cellular adhesion in comparison with the untreated domain, underlying the requirement for a 3D-fold arrangement of the interaction to bind the integrin counterpart: the precise and stable geometry pattern around the flexible loop at the apex of the trimeric structure is essential for the correct interaction of α4β1 integrin with the E933 residue. EMILIN1 presents unique structural features compared to all the other members of the C1q superfamily. While it presents the classical jelly-roll β-sandwich fold, EMILIN1 has only 9 instead of 10 β-strands and this different structural organization is associated with the specific and unique capability to be recognized by α4β1 integrin at an inserted short flexible sequence^18^. It is of note that the present findings of a very specific cleavage site for NE in the gC1q domain of EMILIN1 that profoundly affects the pro-adhesive as well as the proliferation suppressive functions of EMILIN1 depend on a cleavage within the gC1q domain. The cleavage of some members belonging to the gC1q-TNF family has been documented^39^,^40^,^41^,^42^, but almost always outside the globular domain. In particular, adiponectin cleavage by NE in the collagen-like region generates globular fragments with distinct biological activity^40^; the collagen-like region of the C1q subunit of the complement (C1q-CLR) can be cleaved by MMPs^42^; the cardioprotective cardiokine C1q-TNF-related protein-9, requires proteolytic cleavage to obtain a biologically active globular domain isoform^41^; and the differential proteolytic processing of CTRP12 by PCSK3/furin in the N-terminal domain generates functionally distinct isoforms^39^. Only one example of a cleavage site for trypsin located in the globular region of C1q that affected the B chain capability to interact with fucoidan *in vitro* was reported suggesting that the compactness of this domain is very rarely affected by enzymes^43^. To our knowledge, our study demonstrates for the first time the cleavage within the globular domain under physio-pathological conditions of proteolytic process establishing both a unique role of NE as enzyme and a novel specificity for the EMILIN1-gC1q as substrate.

In conclusion, our findings reinforce the uniqueness of the gC1q-α4β1 integrin binding which is highly dependent on the overall structural conformation of the homotrimeric assembly employing a different mode of integrin engagement generally located in mobile loops protruding from the main core of the whole ligand^44^. In this study we demonstrated that only NE is able to disrupt the correct folding of gC1q domain thus impairing its suppressor role on cellular proliferation.

## Materials and Methods

### Antibodies and reagents

Rabbit antiserum against human EMILIN1 (As556) was obtained as previously described^5^,^27^. The antiserum was also purified by affinity chromatography for gC1q recognition (AP As556). EMILIN1, secreted by HEK293 cells, constitutively expressing the EBNA 1 protein, was obtained as previously described^30^. Briefly, the cells were expanded to mass culture and were then maintained for 2 days in serum-free medium to allow accumulation of EMILIN1 in the cell supernatant. Partial purification was achieved by dialysis of the conditioned medium at 4 °C against 0.1 M NaCl, 20 mM Tris-HCI, pH 6.8. A further purification step was achieved by chromatography on a DEAE-cellulose column and size exclusion chromatography using Sepharose CL 4B (Amersham Pharmacia Biotech) as described^30^. The preparation was checked for the presence of laminin and FN by immunoblotting with specific antibodies and proved to be nearly devoid of these contaminants. The C-terminal gC1q domain was produced as previously described^15^,^18^. Plasma FN was purchased from Sigma. Rat tail collagen type I was provided by BD Bioscience. Human NE was provided by Calbiochem, (Merk Millipore); recombinant catalytic domain of MMP-3, MMP-9 and MT1-MMP were purchased by Giotto Biotech Srl. Full length recombinant MMP-9 was provided by Merck Millipore. Collagenase Type I-A and PR3 were provided by Sigma-Aldrich and Enzo LifeScience, Inc., respectively.

### Cell cultures

The human leiomyosarcoma SKLMS1, the immortalized human T lymphocyte Jurkat cell lines were purchased by ATCC and cultured in DMEM supplemented with 10% FCS and antibiotics.

### Enzymatic digestion

FN, EMILIN1 or gC1q and its mutated variants were incubated with the recombinant MMPs or NE at different molar enzyme/substrate ratios for different times as indicated at 37 °C. The digestion with MMP-3, MMP-9, MT1-MMP and collagenase was carried out in a buffer containing CaCl_2_ (10 mM for MMP-9 and MT1-MMP; 5 mM for MMP-3), 150 mM NaCl, 50 mM Tris, pH 7.5. The solution for NE digestion contained 10 mM CaCl_2_, whereas for PR3 a 150 mM NaCl, 50 mM Tris, pH 7.5 buffer was used. Controls were incubated in the appropriate buffer without enzymes. After the indicated times, reactions were stopped by adding 5 × Laemmli buffer, loaded onto 4–20% polyacrylamide gel, and stained in a 0.05% wt/vol Coomassie G-250, 5% v/v glacial acetic acid solution to monitor substrate degradation. In some experiments we analyzed fragmentation pattern by western blotting technique. The nitrocellulose membranes were saturated with TBS buffer (20 mM Tris and 0.15 M NaCl) containing 0.1% Tween-20 (TBST) and 5% non-fat dry milk for 1 h at room temperature and then incubated at 4 °C overnight with primary antibodies against EMILIN1 or gC1q (As556 and AP As556, respectively). After extensive washing in TBST, the membranes were incubated with rabbit HRP-conjugated secondary antibodies (Amersham, GE Healthcare) and then revealed with the ECL Plus chemiluminescence kit (Amersham, GE Healthcare).

### MALDI TOF analysis

To determine the molecular weights of the gC1q NE cleaved fragments, the digestion mixture was separated by SDS-PAGE using a precast, gradient, 4–20% gel (Biorad). After Comassie G-250 staining, selected digestion bands were excised from the gel and submitted to the in-house proteomic facility for the MALDI TOF analyses.

### Site-directed Mutagenesis

Matching the molecular weights of the digestion fragments with those calculated on the basis of the gC1q primary sequence, resulted in two possible cutting sites, located between L912 (P1) and S913 (P1′) or between S913 (P1) and R914 (P1′). Several point mutations of S913 and R914 were generated by site-directed mutagenesis using the overlapping PCR approach^45^. Briefly, in a first PCR round the primer carrying the desired mutation was used in combination with 5′- and 3′-flanking primers to generate two overlapping fragments using as template the wild type EMILIN1 gC1q coding sequence inserted in the expression vector pQE-30 (Qiagen) between the BamHI and KpnI restriction sites. The overlapping fragments were gel-purified and used as templates in a two-step PCR round consisting of 12 elongation cycles in which the overlapping regions work as primers and addition of the 5′- and 3′-flanking primers carrying BamHI and KpnI restriction sites, respectively, followed by 25 amplification cycles. The final products were purified by wizard SV columns (Promega), cut with BamHI and KpnI, and ligated into pQE-30 vector, cut with the same enzymes. Ligation products were transformed in MI5 bacterial strain. The expression constructs containing mutant sequences were verified by DNA sequencing. The oligonucleotides were purchased from Sigma Genosys.

### Purification of recombinant gC1q mutants and SDS-PAGE analysis

Recombinant proteins (WT and mutant gC1q) were expressed as His6-tagged proteins and extracted under native conditions as previously described^30^. The purification of the soluble mutants was performed by affinity chromatography on nickel-nitrilotriacetic acid resin (Qiagen). When resistance to denaturation by heating in the presence of 1% SDS was assayed, the samples were incubated in sample buffer (20 mM Tris-HCl, pH 7.6, 100 mM NaCl, 25 mM EDTA, 1% SDS, 2 mM phenylmethanesulfonyl fluoride, 5 mM N-ethylmaleimide) for 1 h at different temperatures just before gel loading.

### Functional cellular assays

The quantitative cell adhesion assay used in this study is based on centrifugation (CAFCA assay)^46^. 6-well strips of flexible polyvinyl chloride covered with double-sided tape (bottom units) were coated with EMILIN1, gC1q, the mutated variants or their cleavage products (10 μg/ml; unless noted otherwise). Cells were labelled with the vital fluorochrome calcein acetoxymethyl (Invitrogen) and then aliquoted into the bottom CAFCA miniplates, which were centrifuged to synchronize the contact of the cells with the substrate. The miniplates were then incubated for 20 min at 37 °C and were subsequently mounted together with a similar CAFCA miniplate to create communicating chambers for subsequent reverse centrifugation. The relative number of cells bound to the substrate and cells that failed to bind to the substrate was estimated by top/bottom fluorescence detection in a computer-interfaced Infinite M1000 PRO microplate reader (Tecan Group Ltd.). In addition, to quantitatively and qualitatively monitor cell behaviour in real time, we adopted the technology provided by the Real-Time Cell Analyzer dual plate instrument (XCELLigence system, Roche). The strategy is based on continuous quantitative monitoring of cells as they adhere/spread and proliferate by measuring electrical impedance^47^. The change in impedance caused by cell attachment and dependent on the cell number is expressed as the cell index, which is an arbitrary measurement defined as (Rn − Rb)/15, in which Rb is the background impedance of the well measured with medium alone, and Rn is the impedance of the well measured at any time with cells present. Thus, the cell index is a reflection of overall cell number, attachment quality, and cell morphology that can change as a function of time. For adhesion/spreading experiments, the E-plates 96 were precoated with EMILIN1, gC1q, the mutated variants or the cleavage products (10 μg/ml, unless noted otherwise). Cells were then seeded at 20 × 10^3^ cells/well in FCS-free medium and monitored every 3 or 5 min for the time indicated. To monitor cell proliferation, 5% FCS was added to the wells 2 h after plating. Data analysis was performed using Real-Time Cell Analyzer software supplied with the instrument. Experiments were performed in triplicate.

### Statistical analysis

Statistical significance of the results was determined by using the two-tailed unpaired Student’s t test to determine whether two datasets were significantly different. A value of P < 0.05 was considered significant.

## Additional Information

**How to cite this article**: Maiorani, O. *et al*. Neutrophil elastase cleavage of the gC1q domain impairs the EMILIN1-α4β1 integrin interaction, cell adhesion and anti-proliferative activity. *Sci. Rep.* **7**, 39974; doi: 10.1038/srep39974 (2017).

**Publisher's note:** Springer Nature remains neutral with regard to jurisdictional claims in published maps and institutional affiliations.

## Supplementary Material

Supplemental Methods and Figures

## References

[b1] Hynes R. O. The extracellular matrix: not just pretty fibrils. Science 326, 1216–1219 (2009). 19965464 10.1126/science.1176009 PMC3536535

[b2] Watt F. M. & Fujiwara H. Cell-extracellular matrix interactions in normal and diseased skin. Cold Spring Harb. Perspect. Biol. 3, (2011). 10.1101/cshperspect.a005124 PMC3062212 21441589

[b3] Streuli C. H. Integrins and cell-fate determination. J. Cell Sci. 122, 171–177 (2009). 19118209 10.1242/jcs.018945 PMC2714415

[b4] Moreno-Layseca P. & Streuli C. H. Signalling pathways linking integrins with cell cycle progression. Matrix Biol. 34, 144–153 (2014). 24184828 10.1016/j.matbio.2013.10.011

[b5] Doliana R. . EMILIN, a component of the elastic fiber and a new member of the C1q/tumor necrosis factor superfamily of proteins. J. Biol. Chem. 274, 16773–16781 (1999). 10358019 10.1074/jbc.274.24.16773

[b6] Colombatti A. . The EMILIN protein family. Matrix Biol. 19, 289–301 (2000). 10963989 10.1016/s0945-053x(00)00074-3

[b7] Colombatti A. . The EMILIN/Multimerin family. Front Immunol. 2, 93 (2011). 22566882 10.3389/fimmu.2011.00093 PMC3342094

[b8] Ghai R. . C1q and its growing family. Immunobiology 212, 253–266 (2007). 17544811 10.1016/j.imbio.2006.11.001

[b9] Kishore U. . C1q and tumor necrosis factor superfamily: modularity and versatility. Trends Immunol. 25, 551–561 (2004). 15364058 10.1016/j.it.2004.08.006

[b10] Colombatti A., Bressan G. M., Castellani I. & Volpin D. Glycoprotein 115, a glycoprotein isolated from chick blood vessels, is widely distributed in connective tissue. J. Cell Biol. 100, 18–26 (1985). 3880750 10.1083/jcb.100.1.18 PMC2113488

[b11] Colombatti A., Poletti A., Bressan G. M., Carbone A. & Volpin D. Widespread codistribution of glycoprotein gp 115 and elastin in chick eye and other tissues. Coll. Relat Res. 7, 259–275 (1987). 3311601 10.1016/s0174-173x(87)80032-8

[b12] Danussi C. . Emilin1 deficiency causes structural and functional defects of lymphatic vasculature. Mol. Cell Biol. 28, 4026–4039 (2008). 18411305 10.1128/MCB.02062-07 PMC2423131

[b13] Danussi C. . EMILIN1-α4/α9 integrin interaction inhibits dermal fibroblast and keratinocyte proliferation. J. Cell Biol. 195, 131–145 (2011). 21949412 10.1083/jcb.201008013 PMC3187715

[b14] Zacchigna L. . Emilin1 links TGF-beta maturation to blood pressure homeostasis. Cell 124, 929–942 (2006). 16530041 10.1016/j.cell.2005.12.035

[b15] Spessotto P. . beta 1 Integrin-dependent cell adhesion to EMILIN-1 is mediated by the gC1q domain. J. Biol. Chem. 278, 6160–6167 (2003). 12456677 10.1074/jbc.M208322200

[b16] Spessotto P. . EMILIN1 represents a major stromal element determining human trophoblast invasion of the uterine wall. J. Cell Sci. 119, 4574–4584 (2006). 17074837 10.1242/jcs.03232

[b17] Danussi C. . An EMILIN1-negative microenvironment promotes tumor cell proliferation and lymph node invasion. Cancer Prev. Res. (Phila) 5, 1131–1143 (2012). 22827975 10.1158/1940-6207.CAPR-12-0076-T

[b18] Verdone G. . The solution structure of EMILIN1 globular C1q domain reveals a disordered insertion necessary for interaction with the alpha4beta1 integrin. J. Biol. Chem. 283, 18947–18956 (2008). 18463100 10.1074/jbc.M801085200

[b19] Rose D. M., Alon R. & Ginsberg M. H. Integrin modulation and signaling in leukocyte adhesion and migration. Immunol. Rev. 218, 126–134 (2007). 17624949 10.1111/j.1600-065X.2007.00536.x

[b20] Hauzenberger D., Klominek J., Bergstrom S. E. & Sundqvist K. G. T lymphocyte migration: the influence of interactions via adhesion molecules, the T cell receptor, and cytokines. Crit Rev. Immunol. 15, 285–316 (1995). 8834453 10.1615/critrevimmunol.v15.i3-4.60

[b21] Humphries J. D., Byron A. & Humphries M. J. Integrin ligands at a glance. J. Cell Sci. 119, 3901–3903 (2006). 16988024 10.1242/jcs.03098 PMC3380273

[b22] Humphries M. J. The molecular basis and specificity of integrin-ligand interactions. J. Cell Sci. 97(Pt 4), 585–592 (1990). 2077034 10.1242/jcs.97.4.585

[b23] Danussi C. . EMILIN1/alpha9beta1 integrin interaction is crucial in lymphatic valve formation and maintenance. Mol. Cell Biol. 33, 4381–4394 (2013). 24019067 10.1128/MCB.00872-13 PMC3838180

[b24] Kamarajan P., Garcia-Pardo A., D’Silva N. J. & Kapila Y. L. The CS1 segment of fibronectin is involved in human OSCC pathogenesis by mediating OSCC cell spreading, migration, and invasion. BMC. Cancer 10, 330 (2010). 20579373 10.1186/1471-2407-10-330 PMC3146068

[b25] Zucchetto A. . The CD49d/CD29 complex is physically and functionally associated with CD38 in B-cell chronic lymphocytic leukemia cells. Leukemia 26, 1301–1312 (2012). 22289918 10.1038/leu.2011.369

[b26] Lund S. A. . Osteopontin mediates macrophage chemotaxis via alpha4 and alpha9 integrins and survival via the alpha4 integrin. J. Cell Biochem. 114, 1194–1202 (2013). 23192608 10.1002/jcb.24462 PMC12462639

[b27] Pivetta E. . Neutrophil elastase-dependent cleavage compromises the tumor suppressor role of EMILIN1. Matrix Biol. 34, 22–32 (2014). 24513040 10.1016/j.matbio.2014.01.018

[b28] Pivetta E. . Local inhibition of elastase reduces EMILIN1 cleavage reactivating lymphatic vessel function in a mouse lymphoedema model. Clin. Sci. (Lond) 130, 1221–1236 (2016). 26920215 10.1042/CS20160064 PMC4888021

[b29] Stegemann C. . Proteomic identification of matrix metalloproteinase substrates in the human vasculature. Circ. Cardiovasc. Genet. 6, 106–117 (2013). 23255316 10.1161/CIRCGENETICS.112.964452

[b30] Mongiat M. . Self-assembly and supramolecular organization of EMILIN. J. Biol. Chem. 275, 25471–25480 (2000). 10821830 10.1074/jbc.M001426200

[b31] Schilling O. & Overall C. M. Proteome-derived, database-searchable peptide libraries for identifying protease cleavage sites. Nat. Biotechnol. 26, 685–694 (2008). 18500335 10.1038/nbt1408

[b32] Hedstrom L. Serine protease mechanism and specificity. Chem. Rev. 102, 4501–4524 (2002). 12475199 10.1021/cr000033x

[b33] Bank U. & Ansorge S. More than destructive: neutrophil-derived serine proteases in cytokine bioactivity control. J. Leukoc. Biol. 69, 197–206 (2001). 11272269

[b34] Pham C. T. Neutrophil serine proteases: specific regulators of inflammation. Nat. Rev. Immunol. 6, 541–550 (2006). 16799473 10.1038/nri1841

[b35] Mittendorf E. A. . Breast cancer cell uptake of the inflammatory mediator neutrophil elastase triggers an anticancer adaptive immune response. Cancer Res. 72, 3153–3162 (2012). 22564522 10.1158/0008-5472.CAN-11-4135 PMC3397251

[b36] Houghton A. M. . Neutrophil elastase-mediated degradation of IRS-1 accelerates lung tumor growth. Nat. Med. 16, 219–223 (2010). 20081861 10.1038/nm.2084 PMC2821801

[b37] Gregory A. D. . Neutrophil elastase promotes myofibroblast differentiation in lung fibrosis. J. Leukoc. Biol. 98, 143–152 (2015). 25743626 10.1189/jlb.3HI1014-493R PMC4763951

[b38] Gregory A. D., Hale P., Perlmutter D. H. & Houghton A. M. Clathrin pit-mediated endocytosis of neutrophil elastase and cathepsin G by cancer cells. J. Biol. Chem. 287, 35341–35350 (2012). 22915586 10.1074/jbc.M112.385617 PMC3471748

[b39] Wei Z., Lei X., Seldin M. M. & Wong G. W. Endopeptidase cleavage generates a functionally distinct isoform of C1q/tumor necrosis factor-related protein-12 (CTRP12) with an altered oligomeric state and signaling specificity. J. Biol. Chem. 287, 35804–35814 (2012). 22942287 10.1074/jbc.M112.365965 PMC3476250

[b40] Waki H. . Generation of globular fragment of adiponectin by leukocyte elastase secreted by monocytic cell line THP-1. Endocrinology 146, 790–796 (2005). 15528304 10.1210/en.2004-1096

[b41] Yuan Y. . C1q-TNF-related protein-9, a novel cardioprotetcive cardiokine, requires proteolytic cleavage to generate a biologically active globular domain isoform. Am. J. Physiol Endocrinol. Metab 308, E891–E898 (2015). 25783894 10.1152/ajpendo.00450.2014 PMC4436995

[b42] Ruiz S., Henschen-Edman A. H., Nagase H. & Tenner A. J. Digestion of C1q collagen-like domain with MMPs-1,-2,-3, and -9 further defines the sequence involved in the stimulation of neutrophil superoxide production. J. Leukoc. Biol. 66, 416–422 (1999). 10496311 10.1002/jlb.66.3.416

[b43] Tissot B. . Mass spectrometry analysis of the oligomeric C1q protein reveals the B chain as the target of trypsin cleavage and interaction with fucoidan. Biochemistry 44, 2602–2609 (2005). 15709773 10.1021/bi047802h

[b44] Casasnovas J. M., Pieroni C. & Springer T. A. Lymphocyte function-associated antigen-1 binding residues in intercellular adhesion molecule-2 (ICAM-2) and the integrin binding surface in the ICAM subfamily. Proc. Natl. Acad. Sci. USA 96, 3017–3022 (1999). 10077629 10.1073/pnas.96.6.3017 PMC15887

[b45] Cannizzaro R. . Endomicroscopy and cancer: a new approach to the visualization of neoangiogenesis. Gastroenterol. Res. Pract. 2012, 537170 (2012). 22287958 10.1155/2012/537170 PMC3263616

[b46] Spessotto P. . Fluorescence-based assays for *in vitro* analysis of cell adhesion and migration. Methods Mol. Biol. 522, 221–250 (2009). 19247614 10.1007/978-1-59745-413-1_16

[b47] Xing J. Z. . Dynamic monitoring of cytotoxicity on microelectronic sensors. Chem. Res. Toxicol. 18, 154–161 (2005). 15720119 10.1021/tx049721s

