# Supplementary material for: Neutrophil elastase cleavage of the gC1q domain impairs the EMILIN1-α4β1 integrin interaction, cell adhesion and anti-proliferative activity
Source: Sci Rep. 2017 Jan 11;7:39974. doi: 10.1038/srep39974 (PMC5225433; doi:10.1038/srep39974)
Supplement: Supplemental Methods and Figures [file srep39974-s1.pdf]

Supplemental material

Neutrophil elastase cleavage of the gC1q domain impairs the EMILIN1-α4β1 integrin interaction, cell adhesion and anti-proliferative activity

Orlando Maiorani, Eliana Pivetta, Alessandra Capuano, Teresa Maria Elisa Modica, Bruna Wassermann, Francesco Bucciotti, Alfonso Colombatti, Roberto Doliana and Paola Spessotto

Supplemental MATERIALS AND METHODS

Enzymatic digestion using transfected cells. To test the activity of full-extent MMP-3 and MT1-MMP on EMILIN1 we used HEK293 cells as MMP source. HEK293 cells (purchased by ATCC) were transiently transfected with pCMV2-FLAG and pCMV3-SP-FLAG plasmids (Sino Biological Inc), containing flag–tagged MMP-3 and MT1-MMP sequences, respectively, using FUGENE HD transfection reagent (Promega). 24 h after transfection, 150 µg of recombinant EMILIN1 were added to the media (2 ml, total volume). The conditioned media and total cell lysates were collected 48 h post transfection for western blot analysis. Cells were harvested and lysed by vortexing for 15 s in cold HNTG buffer (1% Triton X-100, 20 mM HEPES pH 7.5, 10% glycerol, 150 mM NaCl) containing a protease inhibitors cocktail (Roche) and 1 mM Sodium Orthovanadate followed by incubation on ice for 20 min and centrifugation for 30 min at 16,000 × g at 4 °C. The total protein concentration of the extracts was determined by the Biorad Protein Assay Kit (Biorad) and equal protein loading was confirmed by vinculin detection in whole-cell lysates. Harvested culture media were cleared for detached cells and debris by centrifugation for 10 min at 1,500×g and directly loaded in SDS-PAGE. MMPs expression was probed with anti-flag antibody (Sigma). As MMP-9 source we used supernatants derived by human osteoclast cultures and the relative enzymatic activity was assessed by zymography as previously described [1, 2]. Briefly, monocytes were allowed to grow in the presence of differentiating agents (M-CSF and RANKL, Prepotech) and after 14 days from the beginning of culture the supernatants preincubated in the presence or absence of APMA (p-amino-phenol-mercuric acetate, provided by Sigma-Aldrich) were loaded on a 8% SDS-polyacrylamide gel containing 0.1% gelatin. Gels were run under non-reducing conditions and stained in 0.5% Coomassie brilliant blue R-250/30% methanol/10% acetic acid. After destaining, images of Coomassie blue-stained gels were acquired.

Supplemental Figure S1

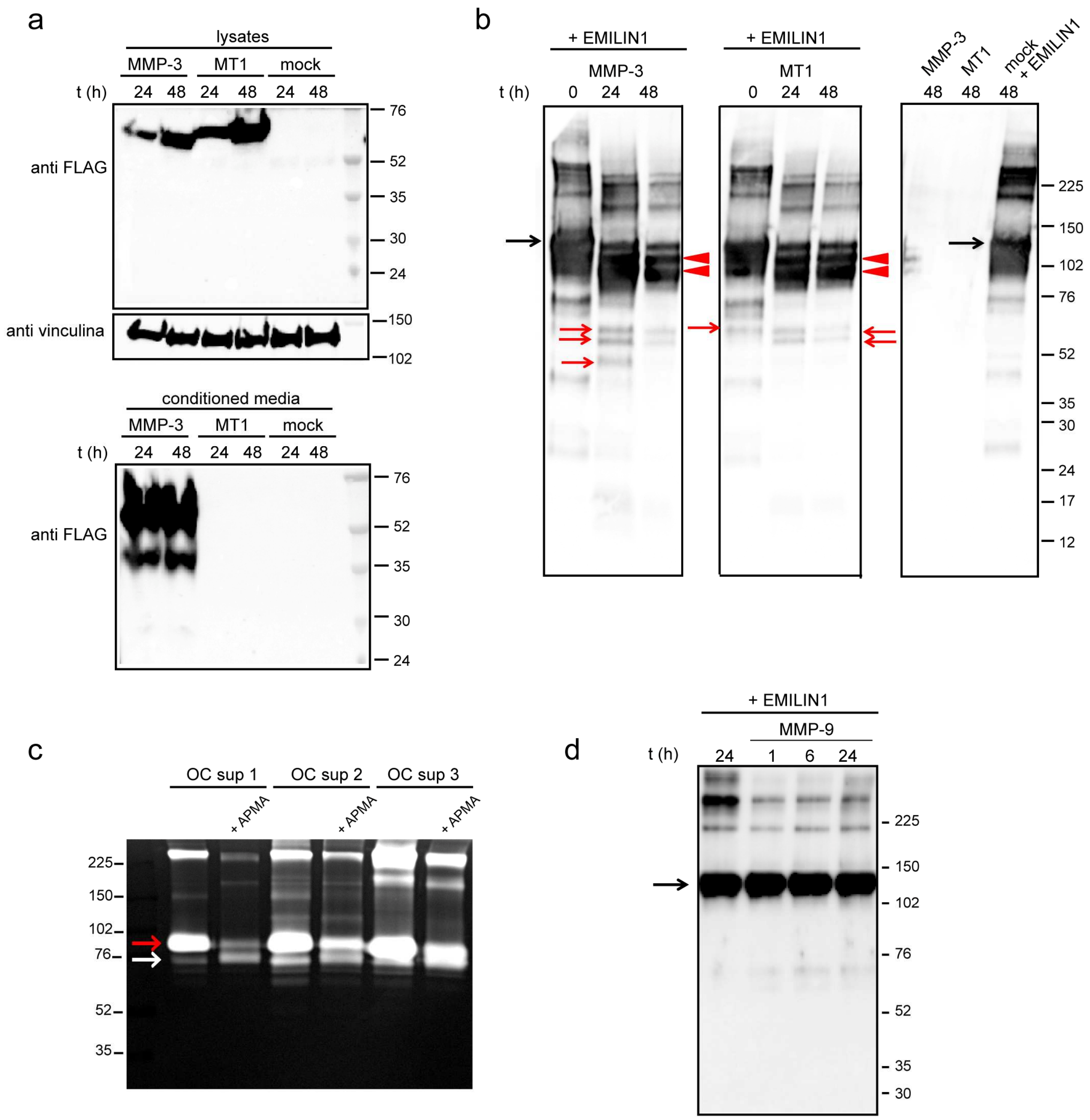

Figure S1. EMILIN1 is partially cleaved by MMPs. (**a**) WB analyses to detect MMP-3 and MT1-MMP (MT1) expression in lysates and supernatants of HEK293 transfected cells. Vinculin was used as loading control. (**b**) EMILIN1 was added to HEK293 24 h post transfection. Supernatants were collected after 24 and 48 h and analysed by WB using anti human EMILIN1 antibody (As556). Red arrow heads indicate two main bands migrating very close to the band corresponding to the intact protein (black arrow). Red arrows indicate cleaved fragments not detectable in the supernatant of mock cells. (**c**) Zymographic analysis showing the presence of

MMP-9 (pro-enzyme, red arrow; active form, white arrow) in three supernatants (sup) obtained from human osteoclast (OC) cultures. APMA was used to activate MMP-9 pro-enzyme. (**d**) EMILIN1, added to OC supernatants, was collected after 1, 6 or 24 h and analysed in WB with As556 antibody to detect cleaved fragments.

Supplemental Figure S2

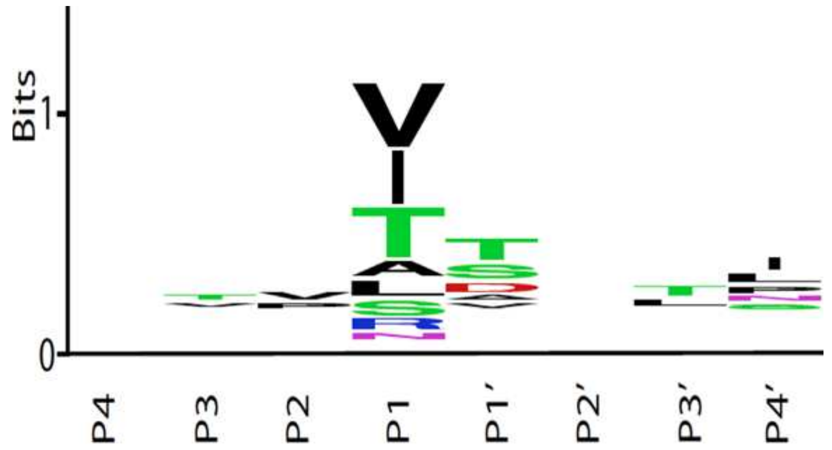

Specificity matrix

Amino acid	P4	P3	P2	P1	P1'	P2'	P3'	P4'
Gly	42	28	51	2	54	60	33	35
Pro	35	3	77	2	3	9	39	64
Ala	37	25	41	61	37	32	28	30
Val	30	27	29	140	23	22	46	23
Leu	33	32	62	32	20	86	59	41
Ile	32	32	24	127	18	31	20	27
Met	10	29	23	2	33	8	10	9
Phe	21	12	19	10	16	39	13	22
Tyr	12	14	5	2	14	18	9	5
Trp	4	1	1	**0**	**2**	14	2	5
Ser	13	28	32	17	68	33	37	28
Thr	19	38	31	54	55	19	21	26
Cys	12	10	6	4	7	8	8	7
Asn	16	22	7	18	28	28	30	37
Gln	14	38	17	2	22	11	24	11
Asp	15	21	4	1	18	11	34	36
Glu	27	69	20	1	8	13	23	26
Lys	6	16	10	1	18	9	26	14
Arg	9	12	10	5	14	13	5	6
His	19	14	9	0	18	12	9	23

Figure S2. Neutrophil elastase site specificity. NE shows a quite loose consensus cleavage site as reported in MEROPS database (http://merops.sanger.ac.uk). **Top**, cleavage site sequence Logo showing the specificity preference in each of the subsites P4-P4'. **Bottom**, specificity matrix showing how frequently each residue has been found to occur in each position around the scissile band. The most conserved residues are at -1 (P1) and +1 (P1') position, but only Histidine and Tryptophan are never allowed in P1, whereas Tryptophan and Proline are very rarely present in position P1'. On the contrary, Serine and Arginine, the residues we indicated as possible cleavage site, are not rarely present in position P1 or in position P1', suggesting that these residues represent a candidate for a NE cleavage site. To experimentally test this hypothesis we prepared several constructs where S913 and R914 were substituted with residues rarely or never present in the predicted cleavage consensus site.

References

1. Pivetta, E. *et al.* MMP-13 stimulates osteoclast differentiation and activation in tumour breast bone metastases. *Breast Cancer Res.* **13**, R105 (2011).

2. Pivetta, E. *et al.* Functional osteoclastogenesis: the baseline variability in blood donor precursors is not associated with age and gender. *Oncotarget.* **6**, 31889-31900 (2015).
